# (2,2′-Bipyridine-κ^2^ *N*,*N*′)iodido(pyrrol­idine-1-dithio­carboxyl­ato-κ^2^ *S*,*S*′)copper(II)

**DOI:** 10.1107/S1600536808008945

**Published:** 2008-04-10

**Authors:** Le-Qing Fan, Ji-Huai Wu

**Affiliations:** a Institute of Materials Physical Chemistry and The Key Laboratory for Functional Materials of Fujian Higher Education, Huaqiao University, Quanzhou, Fujian 362021, People’s Republic of China

## Abstract

In the title compound, [Cu(C_5_H_8_NS_2_)I(C_10_H_8_N_2_)], the Cu^II^ ion is coordinated by one iodide ion, two N atoms of the bipyridine ligand and two S atoms from the pyrrolidine-1-dithio­carboxyl­ate ligand in a distorted square-pyramidal environment.

## Related literature

For related literature, see: Englhardt *et al.* (1998 ▶); Fernández *et al.* (2000 ▶); Koh *et al.* (2003 ▶); Noro *et al.* (2000 ▶); Yaghi *et al.* (1998 ▶).
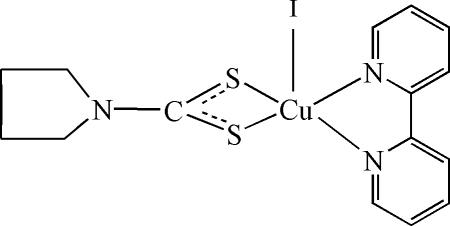


## Experimental

### 

#### Crystal data

[Cu(C_5_H_8_NS_2_)I(C_10_H_8_N_2_)]
*M* *_r_* = 492.87
Monoclinic, 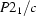

*a* = 6.606 (3) Å
*b* = 16.212 (8) Å
*c* = 16.405 (8) Å
β = 98.399 (10)°
*V* = 1738.3 (15) Å^3^
*Z* = 4
Mo *K*α radiation
μ = 3.27 mm^−1^
*T* = 293 (2) K
0.20 × 0.20 × 0.10 mm

#### Data collection

Rigaku Mercury CCD diffractometer
Absorption correction: multi-scan (*CrystalClear*; Rigaku, 2000 ▶) *T*_min_ = 0.722, *T*_max_ = 1.000 (expected range = 0.521–0.721)
13239 measured reflections
3983 independent reflections
3326 reflections with *I* > 2σ(*I*)
*R*_int_ = 0.029

#### Refinement

*R*[*F* ^2^ > 2σ(*F* ^2^)] = 0.034
*wR*(*F* ^2^) = 0.085
*S* = 1.07
3983 reflections
199 parameters
H-atom parameters constrained
Δρ_max_ = 0.54 e Å^−3^
Δρ_min_ = −0.63 e Å^−3^

### 

Data collection: *CrystalClear* (Rigaku, 2000 ▶); cell refinement: *CrystalClear*; data reduction: *CrystalClear*; program(s) used to solve structure: *SHELXS97* (Sheldrick, 2008 ▶); program(s) used to refine structure: *SHELXL97* (Sheldrick, 2008 ▶); molecular graphics: *SHELXTL* (Sheldrick, 2008 ▶); software used to prepare material for publication: *SHELXTL*.

## Supplementary Material

Crystal structure: contains datablocks global, I. DOI: 10.1107/S1600536808008945/at2555sup1.cif

Structure factors: contains datablocks I. DOI: 10.1107/S1600536808008945/at2555Isup2.hkl

Additional supplementary materials:  crystallographic information; 3D view; checkCIF report

## Figures and Tables

**Fig. 1.**
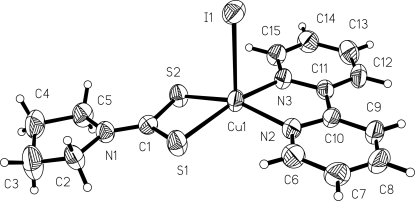
The molecular structure of (I) with 30% probability displacement ellipsoids (arbitrary spheres for H atoms).

## supplementary crystallographic information

### Comment

Research into transition metal complexes has been rapidly expanding because of their fascinating structural diversity, as well as their potential applications as functional materials and enzymes (Noro *et al.*, 2000; Yaghi *et al.*, 1998). Dialkyldithiocarbamates anions, which are typical sulfur ligands, acting as monodentate, bidentate or bridging ligands, are often chosen for the preparation of a considerable structural variety of complexes (Englhardt *et al.*, 1998; Fernández *et al.*, 2000; Koh, *et al.*, 2003). We report here the crystal structure of the title copper(II) complex, (I), contanining a pyrrolidine-1-dithiocarboxylate ligand.

The crystal structure of (I) is built of discrete molecules of the Cu^II^ complex (Fig. 1). The Cu^II^ ion is five-coordinated in a distorted square-pyramidal environment by one I atom in the apical position, two N atoms from the bipyridine ligand and two S atoms from the pyrrolidine-1-dithiocarboxylate ligand in the basal plane (Table 1).

### Experimental

A mixture of Cu(Ac)_2_.H_2_O (0.08 g, 0.4 mmol), NaS_2_CNC_4_H_8_.2H_2_O (0.09 g, 0.4 mmol), 2,2'-bipyridine (0.06 g 0.4 mmol) and NaI.2H_2_O (0.07 g, 0.4 mmol) was stirred in DMF (15 ml). 2-PrOH was diffused into the resulting solution, yielding single crystals of (I).

### Refinement

H atoms were positioned geometrically and refined as riding atoms, with C—H = 0.93 (aromatic) or 0.97 Å (pyrrolidinyl), *U*_iso_(H) = 1.2*U*_eq_(C).

### Crystal data

**Table:**

[Cu(C_5_H_8_NS_2_)I(C_10_H_8_N_2_)]	*F*_000_ = 964
*M**_r_* = 492.87	*D*_x_ = 1.883 Mg m^−^^3^
Monoclinic, *P*2_1_/*c*	Mo *K*α radiation λ = 0.71073 Å
Hall symbol: -P 2ybc	Cell parameters from 1823 reflections
*a* = 6.606 (3) Å	θ = 2.5–27.5º
*b* = 16.212 (8) Å	µ = 3.27 mm^−^^1^
*c* = 16.405 (8) Å	*T* = 293 (2) K
β = 98.399 (10)º	Prism, black
*V* = 1738.3 (15) Å^3^	0.20 × 0.20 × 0.10 mm
*Z* = 4	

### Data collection

**Table:**

Rigaku Mercury CCD diffractometer	3983 independent reflections
Radiation source: Sealed Tube	3326 reflections with *I* > 2σ(*I*)
Monochromator: Graphite Monochromator	*R*_int_ = 0.029
*T* = 293(2) K	θ_max_ = 27.5º
ω scans	θ_min_ = 2.5º
Absorption correction: multi-scan(CrystalClear; Rigaku, 2000)	*h* = −8→8
*T*_min_ = 0.722, *T*_max_ = 1.000	*k* = −20→21
13239 measured reflections	*l* = −21→20

### Refinement

**Table:**

Refinement on *F*^2^	Secondary atom site location: difference Fourier map
Least-squares matrix: full	Hydrogen site location: inferred from neighbouring sites
*R*[*F*^2^ > 2σ(*F*^2^)] = 0.034	H-atom parameters constrained
*wR*(*F*^2^) = 0.085	*w* = 1/[σ^2^(*F*_o_^2^) + (0.041*P*)^2^ + 0.011*P*] where *P* = (*F*_o_^2^ + 2*F*_c_^2^)/3
*S* = 1.07	(Δ/σ)_max_ = 0.001
3983 reflections	Δρ_max_ = 0.54 e Å^−^^3^
199 parameters	Δρ_min_ = −0.63 e Å^−^^3^
Primary atom site location: structure-invariant direct methods	Extinction correction: none

### Special details

**Table:**

Geometry. All e.s.d.'s (except the e.s.d. in the dihedral angle between two l.s. planes) are estimated using the full covariance matrix. The cell e.s.d.'s are taken into account individually in the estimation of e.s.d.'s in distances, angles and torsion angles; correlations between e.s.d.'s in cell parameters are only used when they are defined by crystal symmetry. An approximate (isotropic) treatment of cell e.s.d.'s is used for estimating e.s.d.'s involving l.s. planes.
Refinement. Refinement of *F*^2^ against ALL reflections. The weighted *R*-factor *wR* and goodness of fit *S* are based on *F*^2^, conventional *R*-factors *R* are based on *F*, with *F* set to zero for negative *F*^2^. The threshold expression of *F*^2^ > σ(*F*^2^) is used only for calculating *R*-factors(gt) *etc*. and is not relevant to the choice of reflections for refinement. *R*-factors based on *F*^2^ are statistically about twice as large as those based on *F*, and *R*- factors based on ALL data will be even larger.

### Fractional atomic coordinates and isotropic or equivalent isotropic displacement parameters (Å^2^)

**Table:**

	*x*	*y*	*z*	*U*_iso_*/*U*_eq_	
Cu1	0.47246 (6)	0.43996 (2)	0.34106 (3)	0.03678 (12)	
I1	0.22688 (4)	0.387568 (15)	0.190445 (15)	0.04821 (10)	
S1	0.37645 (15)	0.34944 (6)	0.43793 (6)	0.0475 (2)	
S2	0.74152 (13)	0.34585 (6)	0.36071 (6)	0.0419 (2)	
N1	0.6415 (4)	0.22460 (17)	0.45729 (17)	0.0400 (7)	
N2	0.3012 (4)	0.53917 (17)	0.36333 (17)	0.0383 (6)	
N3	0.6083 (4)	0.52693 (16)	0.28057 (17)	0.0368 (6)	
C1	0.5952 (5)	0.2977 (2)	0.4244 (2)	0.0364 (7)	
C2	0.5177 (6)	0.1803 (2)	0.5103 (2)	0.0485 (9)	
H2A	0.3908	0.1609	0.4789	0.058*	
H2B	0.4865	0.2152	0.5548	0.058*	
C3	0.6532 (9)	0.1087 (3)	0.5431 (4)	0.0826 (16)	
H3A	0.7337	0.1227	0.5955	0.099*	
H3B	0.5716	0.0603	0.5504	0.099*	
C4	0.7884 (8)	0.0936 (3)	0.4791 (3)	0.0711 (13)	
H4A	0.7235	0.0559	0.4373	0.085*	
H4B	0.9180	0.0701	0.5039	0.085*	
C5	0.8212 (6)	0.1769 (2)	0.4417 (3)	0.0512 (10)	
H5A	0.9469	0.2021	0.4682	0.061*	
H5B	0.8262	0.1721	0.3831	0.061*	
C6	0.1427 (6)	0.5395 (2)	0.4055 (2)	0.0497 (9)	
H6A	0.1079	0.4906	0.4297	0.060*	
C7	0.0283 (7)	0.6094 (3)	0.4148 (2)	0.0549 (11)	
H7A	−0.0793	0.6080	0.4455	0.066*	
C8	0.0778 (6)	0.6805 (3)	0.3777 (2)	0.0549 (11)	
H8A	0.0025	0.7283	0.3822	0.066*	
C9	0.2389 (6)	0.6810 (2)	0.3336 (2)	0.0501 (9)	
H9A	0.2730	0.7291	0.3079	0.060*	
C10	0.3503 (5)	0.60970 (19)	0.3277 (2)	0.0369 (7)	
C11	0.5251 (5)	0.60319 (19)	0.2813 (2)	0.0351 (7)	
C12	0.6013 (6)	0.6680 (2)	0.2401 (2)	0.0458 (9)	
H12A	0.5432	0.7202	0.2411	0.055*	
C13	0.7627 (6)	0.6551 (3)	0.1978 (2)	0.0522 (10)	
H13A	0.8167	0.6986	0.1708	0.063*	
C14	0.8441 (6)	0.5771 (2)	0.1955 (2)	0.0497 (9)	
H14A	0.9515	0.5667	0.1661	0.060*	
C15	0.7631 (5)	0.5149 (2)	0.2378 (2)	0.0428 (8)	
H15A	0.8183	0.4622	0.2365	0.051*	

### Atomic displacement parameters (Å^2^)

**Table:**

	*U*^11^	*U*^22^	*U*^33^	*U*^12^	*U*^13^	*U*^23^
Cu1	0.0405 (2)	0.0267 (2)	0.0451 (2)	0.00447 (16)	0.01283 (19)	0.00300 (16)
I1	0.04930 (17)	0.04100 (16)	0.05259 (17)	−0.00012 (10)	0.00166 (12)	−0.00718 (10)
S1	0.0485 (5)	0.0420 (5)	0.0574 (6)	0.0155 (4)	0.0255 (5)	0.0126 (4)
S2	0.0394 (5)	0.0381 (5)	0.0508 (5)	0.0054 (4)	0.0154 (4)	0.0079 (4)
N1	0.0428 (16)	0.0355 (16)	0.0437 (16)	0.0073 (12)	0.0125 (13)	0.0047 (12)
N2	0.0435 (16)	0.0343 (15)	0.0379 (15)	0.0081 (12)	0.0084 (13)	0.0008 (11)
N3	0.0400 (15)	0.0280 (14)	0.0430 (15)	0.0001 (11)	0.0076 (13)	−0.0002 (11)
C1	0.0398 (17)	0.0344 (18)	0.0357 (17)	0.0029 (14)	0.0081 (15)	−0.0011 (13)
C2	0.053 (2)	0.045 (2)	0.049 (2)	0.0033 (17)	0.0125 (18)	0.0156 (16)
C3	0.094 (4)	0.052 (3)	0.107 (4)	0.018 (3)	0.030 (3)	0.031 (3)
C4	0.095 (4)	0.038 (2)	0.083 (3)	0.020 (2)	0.021 (3)	0.009 (2)
C5	0.049 (2)	0.046 (2)	0.061 (2)	0.0199 (17)	0.0170 (19)	0.0053 (18)
C6	0.054 (2)	0.050 (2)	0.048 (2)	0.0113 (18)	0.0144 (18)	0.0037 (17)
C7	0.055 (2)	0.065 (3)	0.047 (2)	0.022 (2)	0.0126 (19)	−0.0027 (18)
C8	0.061 (2)	0.048 (2)	0.054 (2)	0.023 (2)	0.001 (2)	−0.0090 (18)
C9	0.063 (2)	0.034 (2)	0.049 (2)	0.0139 (17)	−0.0024 (19)	−0.0033 (16)
C10	0.0429 (18)	0.0295 (17)	0.0362 (17)	0.0049 (13)	−0.0008 (15)	−0.0031 (13)
C11	0.0372 (17)	0.0292 (17)	0.0363 (17)	−0.0012 (13)	−0.0031 (14)	0.0011 (13)
C12	0.051 (2)	0.0270 (18)	0.057 (2)	0.0008 (15)	0.0004 (19)	0.0038 (15)
C13	0.054 (2)	0.044 (2)	0.059 (2)	−0.0094 (18)	0.010 (2)	0.0128 (17)
C14	0.052 (2)	0.048 (2)	0.052 (2)	−0.0051 (18)	0.0155 (18)	0.0043 (17)
C15	0.0446 (19)	0.037 (2)	0.049 (2)	−0.0012 (15)	0.0147 (17)	−0.0015 (15)

### Geometric parameters (Å, °)

**Table:**

Cu1—N3	2.010 (3)	C4—H4A	0.9700
Cu1—N2	2.030 (3)	C4—H4B	0.9700
Cu1—S1	2.3185 (12)	C5—H5A	0.9700
Cu1—S2	2.3289 (13)	C5—H5B	0.9700
Cu1—I1	2.8789 (11)	C6—C7	1.382 (5)
S1—C1	1.713 (3)	C6—H6A	0.9300
S2—C1	1.712 (3)	C7—C8	1.365 (6)
N1—C1	1.319 (4)	C7—H7A	0.9300
N1—C2	1.466 (4)	C8—C9	1.371 (6)
N1—C5	1.471 (4)	C8—H8A	0.9300
N2—C6	1.337 (5)	C9—C10	1.381 (5)
N2—C10	1.345 (4)	C9—H9A	0.9300
N3—C15	1.336 (4)	C10—C11	1.476 (5)
N3—C11	1.354 (4)	C11—C12	1.384 (5)
C2—C3	1.516 (5)	C12—C13	1.371 (5)
C2—H2A	0.9700	C12—H12A	0.9300
C2—H2B	0.9700	C13—C14	1.377 (6)
C3—C4	1.495 (7)	C13—H13A	0.9300
C3—H3A	0.9700	C14—C15	1.375 (5)
C3—H3B	0.9700	C14—H14A	0.9300
C4—C5	1.511 (5)	C15—H15A	0.9300
			
N3—Cu1—N2	80.44 (12)	C3—C4—H4B	110.5
N3—Cu1—S1	165.74 (8)	C5—C4—H4B	110.5
N2—Cu1—S1	99.36 (9)	H4A—C4—H4B	108.7
N3—Cu1—S2	98.11 (9)	N1—C5—C4	103.4 (3)
N2—Cu1—S2	158.17 (8)	N1—C5—H5A	111.1
S1—Cu1—S2	76.71 (4)	C4—C5—H5A	111.1
N3—Cu1—I1	91.12 (8)	N1—C5—H5B	111.1
N2—Cu1—I1	97.42 (8)	C4—C5—H5B	111.1
S1—Cu1—I1	103.02 (4)	H5A—C5—H5B	109.0
S2—Cu1—I1	104.39 (4)	N2—C6—C7	122.9 (4)
C1—S1—Cu1	84.31 (12)	N2—C6—H6A	118.6
C1—S2—Cu1	84.01 (12)	C7—C6—H6A	118.6
C1—N1—C2	124.5 (3)	C8—C7—C6	118.2 (4)
C1—N1—C5	123.1 (3)	C8—C7—H7A	120.9
C2—N1—C5	112.3 (3)	C6—C7—H7A	120.9
C6—N2—C10	118.6 (3)	C7—C8—C9	119.7 (4)
C6—N2—Cu1	126.6 (3)	C7—C8—H8A	120.2
C10—N2—Cu1	114.8 (2)	C9—C8—H8A	120.2
C15—N3—C11	118.7 (3)	C8—C9—C10	119.7 (4)
C15—N3—Cu1	126.0 (2)	C8—C9—H9A	120.2
C11—N3—Cu1	115.1 (2)	C10—C9—H9A	120.2
N1—C1—S2	122.8 (3)	N2—C10—C9	121.0 (4)
N1—C1—S1	122.4 (3)	N2—C10—C11	114.8 (3)
S2—C1—S1	114.70 (19)	C9—C10—C11	124.1 (3)
N1—C2—C3	103.5 (3)	N3—C11—C12	120.8 (3)
N1—C2—H2A	111.1	N3—C11—C10	114.8 (3)
C3—C2—H2A	111.1	C12—C11—C10	124.4 (3)
N1—C2—H2B	111.1	C13—C12—C11	119.8 (3)
C3—C2—H2B	111.1	C13—C12—H12A	120.1
H2A—C2—H2B	109.0	C11—C12—H12A	120.1
C4—C3—C2	105.0 (4)	C12—C13—C14	119.3 (4)
C4—C3—H3A	110.8	C12—C13—H13A	120.3
C2—C3—H3A	110.8	C14—C13—H13A	120.3
C4—C3—H3B	110.8	C15—C14—C13	118.5 (4)
C2—C3—H3B	110.8	C15—C14—H14A	120.7
H3A—C3—H3B	108.8	C13—C14—H14A	120.7
C3—C4—C5	105.9 (4)	N3—C15—C14	122.8 (4)
C3—C4—H4A	110.5	N3—C15—H15A	118.6
C5—C4—H4A	110.5	C14—C15—H15A	118.6
